# Influenza Hemagglutinin Nanoparticle Vaccine Elicits Broadly Neutralizing Antibodies against Structurally Distinct Domains of H3N2 HA

**DOI:** 10.3390/vaccines8010099

**Published:** 2020-02-22

**Authors:** Alyse D. Portnoff, Nita Patel, Michael J. Massare, Haixia Zhou, Jing-Hui Tian, Bin Zhou, Vivek Shinde, Gregory M. Glenn, Gale Smith

**Affiliations:** Novavax, Inc. Gaithersburg, MD 20878, USA; aportnoff@novavax.com (A.D.P.); npatel@novavax.com (N.P.); mmassare@novavax.com (M.J.M.); hzhou@novavax.com (H.Z.); jhtian@novavax.com (J.-H.T.); bzhou@novavax.com (B.Z.); vshinde@novavax.com (V.S.);

**Keywords:** influenza, hemagglutinin, nanoparticle, vaccine, antibody

## Abstract

Influenza vaccine effectiveness varies annually due to the fast evolving seasonal influenza A(H3N2) strain and egg-derived mutations—both of which can cause a mismatch between the vaccine and circulating strains. To address these limitations, we have developed a hemagglutinin (HA)-based protein-detergent nanoparticle influenza vaccine (NIV) with a saponin-based Matrix-M™ adjuvant. In a phase 1 clinical trial of older adults, the vaccine demonstrated broadly cross-reactive A(H3N2) HA antibody responses. Two broadly neutralizing monoclonal antibodies derived from NIV-immunized mice were characterized by transmission electron microscopy (TEM), antibody competition assays, fluorescence-activated cell sorting (FACS) analysis, and protein–protein docking. These antibodies recognize two conserved regions of the head domain, namely the receptor binding site and the vestigial esterase subdomain, thus demonstrating the potential for an HA subunit vaccine to elicit antibodies targeting structurally and antigenically distinct but conserved sites. Antibody competition studies with sera from the phase 1 trial in older adults confirmed that humans also make antibodies to these two head domains and against the highly conserved stem domain. This data supports the potential of an adjuvanted recombinant HA nanoparticle vaccine to induce broadly protective immunity and improved vaccine efficacy.

## 1. Introduction

Influenza disease burden in the United States is estimated to cause 140,000–960,000 hospitalizations each year and 12,000–79,000 deaths according to the Centers for Disease Control and Prevention (CDC) reporting over the last eight seasons [1]. Despite an increase in vaccination of older adults (≥ 65 years) in the US from 15% to 65% between 1980 and 2001, influenza-related mortality continued to increase [2]. This older adult population accounts for 71–85% of all influenza-related deaths, emphasizing the need for a better vaccine that can overcome the challenges of immune senescence and reduced adaptability of B cell responses to influenza after years of repeated exposure [3,4,5]. Seasonal influenza is caused by co-circulation of influenza A(H1N1), A(H3N2), and influenza B strains. However, seasons dominated by influenza A(H3N2) viruses are more severe, with average pneumonia and influenza mortality rates up to 2.8 times greater than seasons in which A(H1N1) or influenza B strains dominate circulation [2,6]. The rapid evolution of influenza A(H3N2) strains requires continual surveillance of circulating strains to inform annual vaccine recommendations since strain mismatch reduces vaccine efficacy as much as 10% for H3N2 strains [7,8]. To protect against this fast evolving virus, the ideal vaccine antigen would elicit a strong polyclonal antibody response covering a variety of conserved epitopes and antigenic sites on HA, thus reducing the opportunity for escape mutants arising from seasonal antigenic drift to cause severe disease [9].

To date, many broadly neutralizing antibodies (bnAbs) have been isolated and characterized targeting either the highly conserved stem domain or the more variable receptor binding site (RBS) on the head of HA [10,11,12,13,14,15]. Stem-targeting bnAbs can neutralize across subtypes and subgroups, and typically function by blocking the viral fusion machinery, or utilize Fc-mediated effector functions [16]. Head-targeting bnAbs are more restricted within a subtype and often target the receptor binding site (RBS) to block viral attachment. However, more recently, bnAbs binding at or near the vestigial esterase (VE) subdomain located on the side of the head domain of HA have been described, which block viral egress [17] or pH-induced conformational changes [18]. Furthermore, a new subclass of bnAbs isolated from human peripheral blood mononuclear cells (PBMCs) has been found to destabilize the HA trimer [19,20]. One such antibody, FluA-20, recognizes a unique conserved site on the uncleaved (HA0) trimer interface [19]. Thus, naturally occurring protective antibodies function using a multitude of mechanisms and target diverse epitopes on the surface glycoprotein HA.

Efforts toward a universal vaccine emphasize the need to elicit broad and durable immunity to protect against seasonal drift variants while avoiding the introduction of egg-derived mutations, but this goal has not yet been achieved [7,8,21]. In testing our recombinant hemagglutinin nanoparticle vaccine in ferrets, we observed broad neutralization of historical H3N2 strains from the past decade [22]. In a randomized, observer-blinded, comparator-controlled phase 1 clinical trial (ClinicalTrials.gov number NCT03293498) of our trivalent HA protein-detergent nanoparticle vaccine (tNIV) with a saponin-based Matrix-M^TM^ adjuvant, it induced significantly greater HAI antibody responses than the comparator against historical and contemporary A(H3N2) vaccine strains [23]. To further explore this observation of broadly neutralizing A(H3N2) antibodies elicited by tNIV, we characterized two monoclonal antibodies isolated from mice immunized with A/Hong Kong/4801/2014 HA nanoparticle (AHK14 HA NP) [22]. The epitopes of these antibodies were elucidated using antibody competition assays, point mutation analysis, and in silico protein modeling. These antibodies were then used to evaluate the diversity of polyclonal responses in ferrets and older adults from vaccination with tNIV compared to a licensed comparator influenza vaccine.

## 2. Materials and Methods 

### 2.1. Vaccinations and Clinical Materials

The ferret study was performed at IIT Research Institute, Chicago, IL, USA, in accordance with the study protocol approved by the IITRI Institutional Animal Care and Use Committee. IITRI facilities have been fully accredited by the Association for Assessment and Accreditation of Laboratory Animal Care (AAALAC) and operates under an Animal Welfare Assurance (#A3475-01) approved by the National Institutes of Health (NIH) Office of Laboratory Animal Welfare (OLAW). Ferret immunization and serum samples were previously described [22]. 

Human serum samples tested were each chosen randomly from volunteers enrolled in the phase 1/2 clinical trial (NCT03293498) from 2017 as previously described [23]. Study cohorts included individuals of both genders, ≥60 years of age, randomized into treatment groups. The clinical trial was a randomized, observer-blinded, active-controlled trial conducted at multiple sites in the US. Informed consent was obtained from all participants. The trial was approved by an institutional review board (Copernicus Group IRB) and conducted in accordance with the Declaration of Helsinki and International Conference on Harmonization Guidelines for Good Clinical Practice.

### 2.2. Culture of Influenza Viruses

Viruses were obtained from the Centers for Disease Control and Prevention, Atlanta, GA, USA. Viruses strains used for the neutralization assay were grown and passaged in MDCK cells except A/Wisconsin/19/17 that was grown in MDCK-SIAT cells. Viruses used in the HAI assay were grown in specific pathogen-free 10-day-old embryonated hen eggs (Charles River, Wilmington, MA, USA). Viruses used in the neutralization assay had their HA gene sequenced to confirm it contained the wild-type genotype and did not contain any egg adapted mutations. Wild-type and egg passage HA sequences were compared to published sequences in the GISAID EpiFlu database.

### 2.3. Neutralization Assays

Virus neutralizing activity was determined using a WHO-recommended neutralization assay [24]. After mAb samples were serial diluted in 96-well microplates, 100 TCID50 of influenza virus was added and incubated at 37C with 5% CO_2_ for 2 h before adding MDCK cells to assay plates. After 18–22 h, MDCK cells were fixed with 80% cold acetone and incubated with murine monoclonal anti-influenza A nucleoprotein (1:1 mixture of MAB8257 and MAB8258, Millipore Billerica, MA, USA) followed by peroxidase-conjugated goat anti-mouse IgG (Kirkegaard and Perry Laboratories, Gaithersburg, MD, USA). Optical density following development with 3, 3′, 5, 5′-tetramethylbenzidine (TMB) substrate (Sigma Aldrich, Saint Louis, MO, USA) was used to calculate the 50% neutralization titer (IC50) for each sample.

### 2.4. Electron Microscopy (TEM and cryoEM) and 2D Class Averages

Electron microscopy was performed by NanoImaging Services (San Diego, CA, USA) with an FEI Tecnai T12 electron microscope operating at 120 keV equipped with an FEI Eagle 4k × 4k CCD camera. HA NP samples were imaged over a layer of continuous carbon supported by nitro-cellulose on a 400 mesh copper grid. Negative-stain EM grids were prepared by applying 3 μL of sample suspension to a cleaned grid, blotting away with filter paper, and immediately staining with uranyl formate. For antibodies bound to HA, Fab fragments were generated using the Pierce^TM^ Mouse IgG_1_ Fab and F(ab’)_2_ micro preparation kit (Thermo Scientific, Waltham, MA, USA) and incubated with HA NP at a molar ratio of 1:10 (HA:Fab) for 30 min at room temperature prior to imaging. CryoEM samples were preserved in vitrified ice supported by holey carbon films in 400 mesh copper grids. Each sample was prepared by applying a 3 uL drop of sample suspension to a cleaned grid, blotting away with filter paper, and immediately proceeding with vitrification in liquid ethane. Grids were stored under liquid nitrogen until transferred to the electron microscope for imaging. The 2D class averaging analysis was performed by first selecting individual particles in the 67,000× high magnification images using automated picking protocols [25] and manual picking. A reference-free alignment strategy based on the XMIPP [26] processing package was used to align selected particles and sort them into self-similar groups of classes.

### 2.5. Cloning, Expression, and purification of Wild-Type and Mutant Hemagglutinins

Influenza virus HA wild-type sequences were taken from published sequences in the GISAID EpiFlu database and accession numbers are listed in Supplemental Table S1. As previously reported, HA genes were codon optimized for high-level expression in *Spodoptera frugiperda* (Sf9) insect cells and synthesized by GenScript (Piscataway, NJ, USA) [22]. HA genes were cloned into pBac1 baculovirus transfer vectors (Millipore Sigma, Billerica, MA, USA) and co-transfected into Sf9 cells with the *flash*BAC^TM^ GOLD system (Oxford Expression Technologies, Oxford, UK) using X-tremeGENE HP transfection reagent (Roche, Indianapolis, IN, USA). Point mutations in the HA gene were made with the QuikChange Lightning site-directed mutagenesis kit (Agilent, Santa Clara, CA, USA). Recombinant HA was purified from Sf9 cell membranes with a combination of ion exchange chromatography, affinity chromatography, tangential flow, and nanofiltration as previously described [22].

### 2.6. Sf9 Expressed HA Fluorescence-Activated Cell Sorting (FACS) Staining

*Spodoptera frugiperda* (Sf9) cells were infected with recombinant baculoviruses with the indicated influenza HA transgene at an MOI of 0.001. Forty-eight hours later, cells were collected and labelled with primary human antibody CR8020 (Creative Biolabs, Shirley, NY, USA), for expression gating, and either A2.91.3 or A.2.4.1 mouse antibodies. Secondary antibodies, PE-conjugated goat anti-human (Southern Biotech, Birmingham, AL) and APC conjugated donkey anti-mouse (Jackson Immunology, West Grove, PA, USA), were used to detect binding of the primary antibodies. The samples were processed using a LSR-Fortessa flow cytometer (Becton Dickinson, San Jose, CA, USA). Data were analyzed using FlowJo software version 10 (Becton, Dicinson & Company, Ashland, OR, USA). 

### 2.7. Epitope Mapping and Competition Binning Analyses by Bio-Layer Interferometry (BLI)

Epitope mapping and competitive binning assays were performed using Octet QK384 (BLI) with amine coupled A/Hong Kong/4801/2014 HA nanoparticle onto amine-reactive biosensor tips (Forté Bio, Molecular Devices, San Jose, CA, USA). Commercial antibodies F045-092, F005-126, and CR8020 (Creative Biolabs, Shirley, NY, USA) and hybridoma derived murine antibodies were bound sequentially (5 μg/mL) for 300 sec each and binding normalized to buffer control without competition (100%). Data analysis was performed with Octet Data Analysis HT 10.0 software using the Epitope Binning.

As previously described, BLI was used to evaluate immune sera for antibodies that cross-competed with the hybridoma isolated murine mAbs, A2.91.3 and A2.4.1, and the stem mAb, CR8020 [27]. Competitive antibody equivalents (CAEs) were measured by Octet QK384 with immobilized homologous A/Hong Kong/4801/14 HA or A/Singapore/INFIMH-0019/2016 HA nanoparticle onto AR2G amine-reactive biosensor tips (Forté Bio, Molecular Devices, San Jose, CA, USA). HA protein biosensor tips were first exposed to immune serum for 300 sec followed by dipping the tips in the competing monoclonal antibody (5 μg/mL) for 300 sec. Assays were performed at 30 ºC with continuous agitation at 1000 rpm. CAE was calculated based on the percentage of competition and the concentration of the mAb. Binding was normalized to buffer control and percentage of binding and competition calculated. Data analysis was performed with Octet Data Analysis HT 10.0 software and CAE in serum samples calculated based on the percentage competition and concentration of mAb. 

### 2.8. Recombinant Antibody Cloning, Expression, and Purification

Murine IgG1 heavy and light chains were codon optimized for mammalian expression and synthesized by GenScript (Piscataway, NJ, USA). Two separate plasmids were made in pcDNA^TM^ 3.4 under control of the CMV promotor, with the murine IgG kappa signal peptide, Woodchuck Post-transcriptional Regulatory Element (WPRE) and TK polyadenylation signal (Invitrogen, Carlsbad, CA, USA). Co-transfection was performed using the Expi293F Expression system according to the manufacturer’s instructions (Gibco, Carlsbad, CA, USA). Secreted antibody was harvested from supernatants and purified using a HiTrap Protein G HP column (GE Healthcare, Chicago, IL, USA).

### 2.9. Hemagglutinin and Antibody Modeling and Protein–Protein Docking

A homology model of A/Hong Kong/4801/2014 HA was built on the X-ray structure of A/Victoria/361/2011 (PDB 4WEA) using the Homology Modeler application in MOE 2018.0101 [28,29]. Three individual HA protomers were modeled maintaining the HA0 structure since HA nanoparticles produced in Sf9 cells are not cleaved into HA1 and HA2 [22]. The extracellular domain sequence template for A/Hong Kong/4801/2014 was 98.2% identical to A/Victoria/361/2011 containing nine amino acid changes. Notably the change from Lys160 to Thr160 added a new glycosylation site at Asn158. The individual HA protomers were then superposed on the A/Victoria/361/2011 HA trimer co-crystallized with Fabs (PDB 4O5I) [30]. Glycans included in the X-ray crystal structure were detached using Protein Builder and reattached to the A/Hong Kong/4801/2014 homology model using Molecule Builder and the new glycan at Asn158 was built using the Carbohydrate Builder tool. All glycans and modified Asn residues were energy minimized with the Amber10:EHT forcefield in MOE 2019.01 (Chemical Computing Group, Montreal, QC, Canada). 

A homology model of the variable region of A2.91.3 was created using the Antibody Modeler application in MOE 2018.01 as previously described [31]. Based on the homology search, PDB 1CIC was used as the framework template with PDBs 5I66, 3V6O, 3UJT for the light chain CDRs and PDBs 1A6V, 1MNU, 4JN2 for the heavy chain CDRs 1, 2, and 3 respectively. 

Protein–protein docking was used to model the Fv A2.91.3 binding to the head domain of HA. To minimize the impact of branched glycan flexibility, all glycans were trimmed down to 4GlcNAcβ1-N-Asn prior to protein–protein docking. The HA trimer was also truncated to exclude the stem region and reduce computation time. Docking simulations were performed with the Receptor defined as the truncated HA trimer and site targeted as the shallow base of the RBS (Y98, W153, and Y195). The A2.91.3 Fv domain was defined as the ligand with the HC CDR3 residues (YYYYD) used for the ligand site. Resulting complexes were analyzed using MOE’s Database Viewer, Protein-Ligand Interaction Fingerprint (PLIF) calculation, and Epitope binning [28].

## 3. Results

### 3.1. Broadly Neutralizing Monoclonal Antibodies Bind the Receptor Binding Site and Vestigial Esterase Subdomain of HA

**Figure 1:**
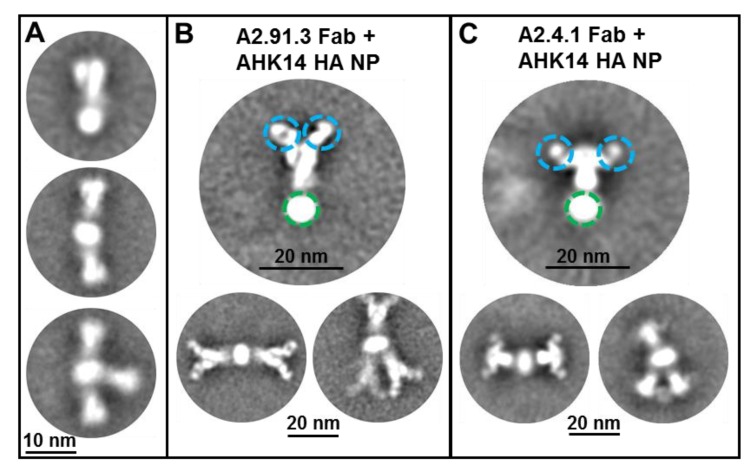
Transmission electron microscopy (TEM) of hemagglutinin (HA) nanoparticles with Fab binding. (**A**) Negative-stain TEM 2D class average images of A/Hong Kong/4801/2014 HA nanoparticles shown with one to three HA trimers per nanoparticle as previously reported [22]. A/Hong Kong/4801/2014 HA nanoparticles were incubated with 1:10 molar ratio of A2.91.3 (**B**) or A2.4.1 (**C**) Fabs prior to imaging. Individual particles were selected using automated and manual picking for 2D class averaging. Fab domains are circled in blue and the detergent core is circled in green.

**Figure 2:**
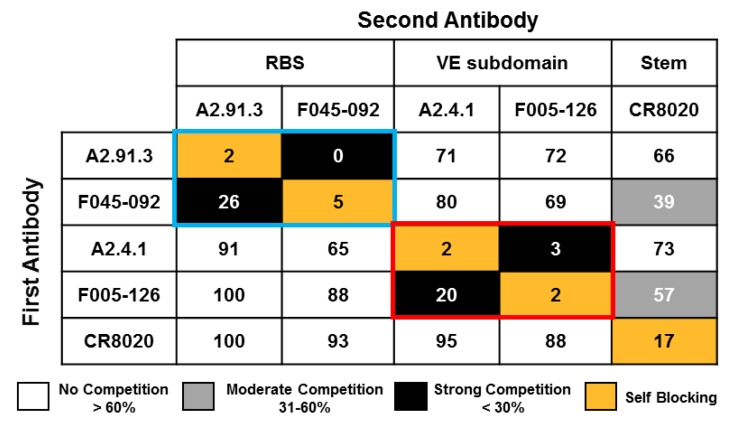
Epitope mapping of A2.91.3 and A2.4.1 by competition with a panel of HA site-specific monoclonal antibodies. Competition binding assays were performed by Octet QK384 Bio-layer interferometry (BLI) with A/Hong Kong/4801/2014 HA nanoparticles amine coupled to biosensor tips. Antibodies were bound sequentially, and binding normalized to buffer control without competition (100%). A2.91.3 had strong competition with receptor binding site (RBS) antibody F045-092 (blue box). A2.4.1 had strong competition with vestigial esterase (VE) subdomain antibody F005-126 (red box). Minimal competition was detected with stem antibody CR8020. Strong competition was defined as < 30% max signal, moderate competition was between 31% and 60%, and no competition was >60% max signal.

Two broadly neutralizing murine monoclonal antibodies (mAbs, A2.91.3 and A2.4.1) were previously identified from immunization with A/Hong Kong/4801/2014 HA nanoparticles (AHK14 HA NP) with Matrix-M^TM^ [22]. To identify the domains of HA targeted by the two isolated mAbs, negative-stain TEM of AHK14 HA NP alone was compared to TEM of AHK14 HA NP incubated with either A2.91.3 or A2.4.1 Fab domains (Figure 1). Both antibodies bound the head domain of HA, but A2.91.3 bound the top (Figure 1B) and A2.4.1 bound the side (Figure 1C) of the head domain. Based on the distinct binding angles of the Fabs with the head region of HA, two structurally characterized broadly neutralizing HA head antibodies (F045-092 and F005-126) were used to further elucidate the epitopes of HA recognized by A2.91.3 and A2.4.1 [32]. The bnAb F045-092 was co-crystallized with A/Victoria/3/1975 and A/Victoria/361/2011 HAs and utilizes its long (23 amino acid) HC CDR3 loop to insert into the RBS and mimic sialic acid binding [30]. F045-092 was used as a representative of the collection of RBS targeting bnAbs characterized to date such as C05, 8F8 (H2 HA), and CH65 (H1 HA) [11,12,13]. There is also a growing group of bnAbs that recognize unique epitopes on the head domain of HA1 at or near the vestigial esterase (VE) subdomain, including F005-126, HC45, CR8071 (Influenza B), H5M9 (H5 HA), and H3v-47 [14,18,33,34,35]. Notably the VE subdomain covers most of antigenic site E and some of antigenic site C, while the RBS encompasses antigenic sites A and B. The bnAb F005-126 binds a cleft formed by two HA monomers and the highly conserved N285 glycan in which the right side of the cleft includes the VE subdomain [18]. Competitive binding of A2.91.3 and A2.4.1 with F045-092, F005-126, and stem antibody CR8020 [15] revealed that A2.91.3 specifically competes with RBS antibody F045-092 and A2.4.1 specifically competes with VE subdomain antibody F005-126 (Figure 2). As expected from the TEM images, neither of these antibodies showed significant competition with the stem antibody CR8020. 

### 3.2. Both Antibodies Bind to Most Circulating H3N2 subclades from the 2017–2018 Season 

**Figure 3:**
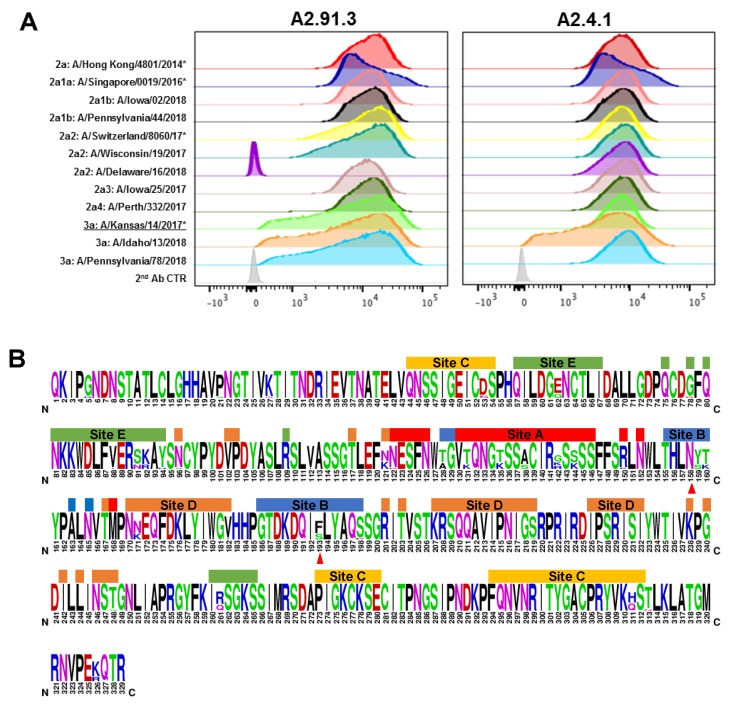
Fluorescence-activated cell sorting (FACS) analysis of antibodies binding to a panel of contemporary H3N2 HAs. (**A**) Sf9 cells were infected with recombinant baculovirus with the H3N2 HA transgene at an MOI of 0.001 per cell for 48 h. Post-infection Sf9 cells were co-labeled with stem antibody CR8020 for gating of HA expression and either A2.91.3 or A2.4.1. Staining with RBS A2.91.3 and VE subdomain A2.4.1 showed differential binding of these antibodies to contemporary H3N2 strains with various mutations in the HA protein. * Indicates WHO H3N2 vaccine strains. (**B**) Sequence logo of HA1 for the 12 contemporary H3N2 strains tested by FACS. Amino acids shown at full height were conserved across all tested strains versus amino acid diversity shown by percentage related to total height. Antigenic sites were labeled according to Munoz et al. [36] and the N158 glycosylation site and F193S mutation site were marked with red triangles.

The seasonal antigenic drift of circulating H3N2 strains is a major contributor to influenza-related illness and hospitalization [8]. The two monoclonal antibodies isolated from immunization with AHK14 HA NP were tested for their ability to recognize circulating strains from the 2017–2018 season and recent vaccine strains (A/Singapore/0019/2016, A/Switzerland/8060/2017, and A/Kansas/14/2017). Recombinant baculoviruses containing various influenza HA genes were made using GISAID EpiFlu sequences as reported in Supplemental Table S1. Figure 3A shows the relative binding of A2.91.3 and A2.4.1 as analyzed by FACS staining of Sf9 cells expressing a collection of contemporary HAs. The strains evaluated cover the diversity of H3N2 subclades of circulating in 2018 (2a1, 2a1a, 2a1b, 2a2, 2a3, 2a4, and 3a) and demonstrate that these two antibodies maintain binding recognition of all strains except A/Delaware/16/18 HA to which A2.91.3 had a loss of binding. Each of the tested strains contained between three and eighteen mutations when compared to A/Hong Kong/4801/2014 HA and mutations were dispersed across all five antigenic sites as shown in Figure 3B [36,37,38].

**Figure 4:**
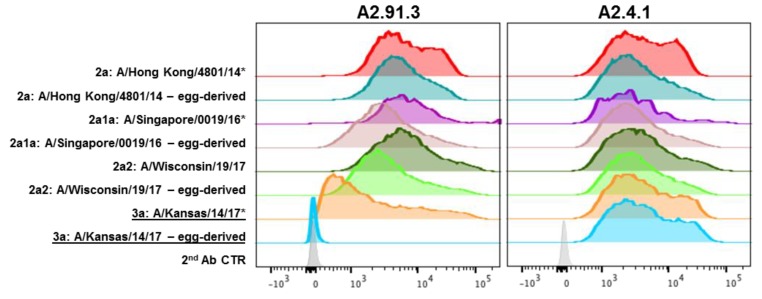
FACS analysis of recent wild-type and egg-passaged H3N2 strains. HA genes from 2014 to 2017 H3N2 strains were cloned into pBac1 baculovirus transfer vectors. Egg-passaged mutations as identified from GISAID EpiFlu sequences were incorporated into each HA gene using QuikChange mutagenesis. Sf9 cells infected with HA containing recombinant baculovirus were co-labeled with stem antibody CR8020 for gating of HA expression and A2.91.3 or A2.4.1. Staining with RBS A2.91.3 or VE subdomain A2.4.1 showed the potential impact of egg-passaged mutations or antigenic drift to the HA head domain. * Indicates WHO H3N2 vaccine strains.

In addition to seasonal antigenic drift, vaccine mismatch due to egg-passaged mutations that arise during manufacturing can also reduce vaccine effectiveness [39,40,41]. Using a recombinant protein vaccine maintains the wild-type HA sequence without risk of mutation during production. To evaluate the impact of egg-passaged mutations, both monoclonal antibodies were tested for binding by FACS (Figure 4) and for neutralization of the wild-type and egg-passaged viruses (Table 1). Both antibodies maintained binding and neutralization of all wild-type H3N2 viruses, though A2.4.1 demonstrated more moderate neutralization potency compared to A2.91.3 as has been observed for other VE targeting antibodies [34]. However, A/Kansas/14/17 with egg-passaged mutations D190N and N246T in HA lost binding and neutralization mediated by RBS antibody A2.91.3 (Figure 4 and Table 1). These two egg-derived mutations significantly alter the head-domain of HA as D190N alters the highly conserved 190 position which has only been Asp or Glu since 1968, and adds to the F193S mutation in the 190-helix of the RBS found in 3C.3a strains [29]. The second egg-derived mutation, N246T, removes the highly conserved glycan at N246 that has persisted since the 1980s [29]. These egg-derived mutations add to the already divergent 3C.3a subclade which compared to recent H3N2 vaccine strains has lost the newest glycosylation site at N158 and incorporated the 130 loop mutation A138S shown to increase red blood cell avidity in HAI assays [42]. Notably wild-type A/Kansas/14/17 has six mutations in antigenic sites A and B surrounding the RBS compared to the most recent vaccine strain A/Singapore/INFIMH-16-0019/2016.

### 3.3. A2.91.3 Binds the RBS at the 190-Helix 

**Figure 5:**
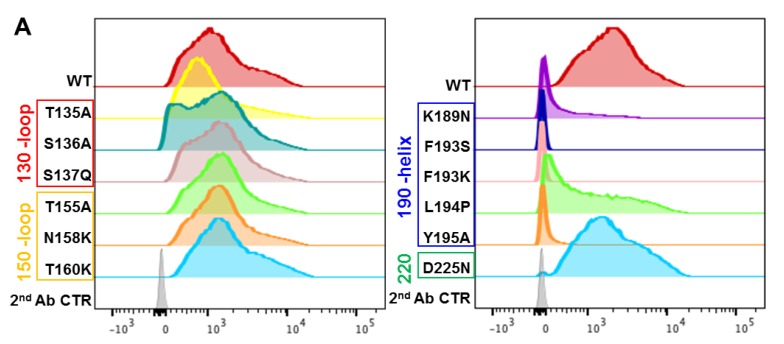

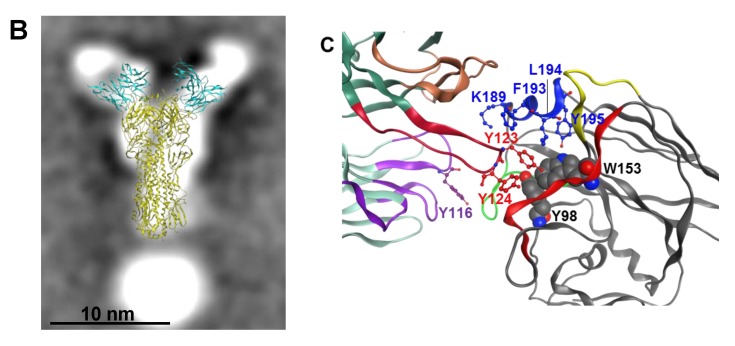
Effect of HA RBS point mutations on A2.91.3 binding determined by FACS analysis. (**A**) AHK14 HA mutants were evaluated by Sf9 expression and FACS dual staining with stem antibody CR8020 for gating of HA expression and A2.91.3 labeling for binding analysis. Mutations in the 190-helix show a significant loss of binding to RBS antibody A2.91.3. (**B**) The antibody angle of approach as visualized by TEM 2D class averaging images was used to screen the models of A2.91.3 Fv binding to the RBS of AHK14 HA. (**C**) The model of A2.91.3 binding to the RBS identified the HC CDR3 loop (red) interacting directly with the RBS pocket. The HC CDR2 (orange) and CDR3 (red) are in contact with the 190-helix, and LC CDR3 loop (purple) interacts with the 220 loop (green) of the RBS and neighboring protomer glycans.

To further detail the epitope in the RBS used by A2.91.3, a collection of single point mutations were made in the A/Hong Kong/4801/2014 HA sequence based on reverting to historical amino acids, disrupting the hydrophobic pocket of the RBS, and published escape mutants for bnAbs in the RBS [43]. FACS analysis of these single point mutations revealed that A2.91.3 interacts directly with the 190-helix near the RBS and a major component of antigenic site B (Figure 5A). Additional mutations in the bottom of the receptor binding site pocket (Tyr98Ala and Trp153Ala) demonstrated that A2.91.3 interacts with the hydrophobic pocket like many bnAbs that utilize sialic acid mimetic binding to the RBS (Figure S1) [11,13,30]. Hybridoma sequencing of A2.91.3 was used to build an Fv model of A2.91.3 using the MOE software Antibody Modeler (Figure S2A) and revealed that the HC CDR3 contained four consecutive Tyr residues. This Fv was docked to a homology model of A/Hong Kong/4801/2014 HA0 trimer built based on A/Victoria/361/2011 (PDB 4WEA). Docking results were evaluated for their agreement with the TEM 2D class averaging images (Figure 5B), their use of HC CDR3, and interaction with the 190-helix of HA. Notably, attempts to dock A2.91.3 binding to the RBS without glycans modeled on the surface yielded dramatically different results than when a single GlcNAc was included at known glycosylation sites (Figure S2B and S2C). The best modelled epitope identified by protein–protein docking utilized the HC CDR3 loop to interact with the RBS pocket (Figure 5C). From the model, HC CDR2 and CDR3 were both in contact with the 190-helix, and LC CDR3 loop interacted with the 220 loop of the RBS and neighboring protomer glycans. Based on the predicted paratope of A2.91.3, single alanine substitutions were tested for binding to A/Hong Kong/4801/2014 HA by bio-layer interferometry (BLI). The single point mutation Y116A in the LC CDR3 significantly reduced binding and when combined with HC CDR3 Y123A mutation completely lost binding to HA (Figure S3).

In addition to the single point mutation analysis of HA, the egg-passaged double mutation T160K/L194P found in A/Hong Kong/4801/2014 was tested for binding to A2.91.3. Interestingly A2.91.3 recovered binding to T160K/L194P egg-derived mutant compared to the L194P mutation alone in A/Hong Kong/4801/2014 HA (Figure S4). This corroborated the importance of glycans identified from modeling A2.91.3 binding to the head domain of HA as the T160K mutation results in a loss of the glycan at N158. It may also explain how A2.91.3 was able to bind and neutralize A/Kansas/14/17 (Figure 4 and Table 1) which contained the F193S mutation but had also lost the N158 glycan due to a reversion at T160K (not associated with egg-derived mutations). Conversely, A2.91.3 lost binding to A/Delaware/16/18 HA which maintained the N158 glycan and had the F193S mutation in the 190-helix (Figure 3A). Thus, RBS-specific antibodies are especially susceptible to the accumulation of both egg-derived and viral escape mutations acquired in the immunodominant antigenic site B [44]. 

### 3.4. Epitope Mapping of VE mAb with Modeling and Point Mutation Analysis

**Figure 6:**
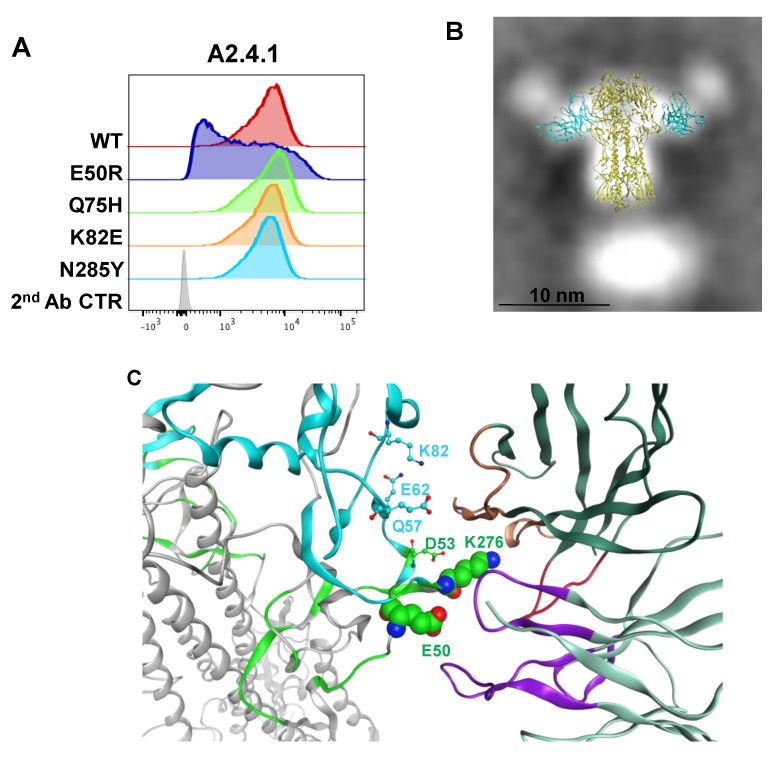
FACS analysis of point mutations in HA and modeling the A2.4.1 epitope. (**A**) Mutations made in AHK14 HA were evaluated by Sf9 expression and FACS dual staining with stem antibody CR8020 for gating of HA expression and A2.4.1 labeling for binding analysis as described in Figure 5. (**B**) The antibody angle of approach as visualized by TEM 2D class averaging images was used to screen the models of A2.4.1 Fv binding to the VE subdomain of AHK14 HA NP. (**C**) Protein–protein docking model of A2.4.1 binding used all six CDRs (LC CDRs, purple; HC CDRs 1&2 orange; HC CDR3, red). A2.4.1 binds antigenic site C (green) at K276, E50, and D53. VE subdomain (cyan) amino acids D53, Q57, E62, and K82 were also involved in docking.

The VE subdomain antibody A2.4.1 had broad neutralization (Table 1) and binding of all the contemporary circulating H3N2 strains tested (Figure 3A). Mutational analysis of A2.4.1 revealed that its binding does not rely upon the glycan at N285 critical to the epitope of F005-126 [18] nor K82 central to the epitope of VE antibody H3v-47 [33] (Figure 6A). Two additional single point mutations near the VE subdomain (E50R and Q75H) identified from historical H3N2 strains were tested with A2.4.1 and revealed the importance of binding at E50 in antigenic site C (Figure 6A). This information was used to model A2.4.1 and evaluate protein–protein docking targeted to interact with amino acid E50 in AHK14 HA. The hybridoma sequencing of A2.4.1 Fv revealed a relatively short HC-CDR3 of only nine amino acids and docking of A2.4.1 to AHK14 HA which aligned to TEM data (Figure 6B) utilized all six CDRs to bind antigenic site C and the lower region of the VE subdomain (Figure 6C). The best fit model surrounds the highly conserved and prominent Lys276 with all three HC CDRs and LC CDR3 amino acids. The HC CDR2 interacts with the VE subdomain at amino acids Asp53, Gln57, Glu62 and Lys82. From the point mutation analysis E50R in HA reduces binding, and in the docked model of A2.4.1 this mutation would create a clash with HC CDR His31 and Ser32. The A2.4.1 docked model also overlaps the location of the VL domain of F005-126 corroborating the antibody competition observed (Figure 2). 

### 3.5. Antibodies Elicited by Immunization of Older Adults Cover Structurally and Antigenically Distinct Domains 

**Figure 7:**
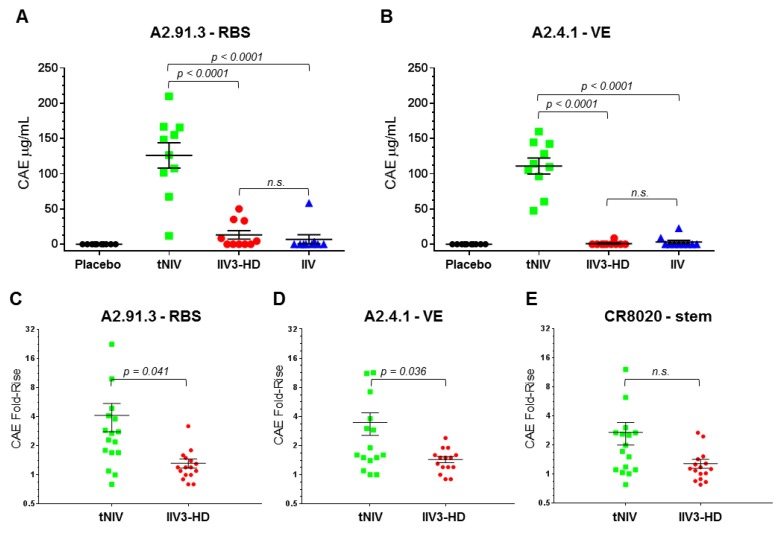
Competing antibodies in the sera of ferrets and older adults. (**A**,**B**) Age- and body weight-matched ferrets were immunized twice at a 3 week interval with 10 animals per group (placebo [PBS]; tNIV [15 mg/strain + 50 mg Matrix-M]; Fluzone HD [IIV3-HD, 60 mg/strain]; or Fluzone [IIV, 15 mg/strain]). Serum collected 3 weeks after the second dose (day 42) was tested for the presence of competing antibodies to the RBS A2.91.3 (**A**) or VE subdomain A2.4.1 (**B**). (**C**,**D**,**E**) Sixteen individuals each were chosen randomly from clinical trial groups (tNIV [60 mg/strain + Matrix-M]; or Fluzone HD [IIV3-HD, 60 mg/strain]). Serum samples were evaluated for the presence of antibodies that compete for binding with monoclonal antibodies to the RBS A2.91.3 (**C**), VE subdomain A2.4.1 (**D**), or stem CR8020 (**E**). Competitive antibody equivalents (CAEs) were measured by Octet QK384 with immobilized A/Hong Kong/4801/2014 HA and fold-rise was calculated as the ratio from pre-immunization (day 0) to post-immunization (day 21) for each sample in the vaccination groups. Individual data points were superimposed by the arithmetic mean and SEM for each group. For samples below the limit of detection (LOD), values were set at half the LOD (0.5 μg/mL). P-values were calculated using the Student’s t-test with *p* < 0.05 reported and *p* > 0.05 indicated as not significant (n.s.).

Classical hemagglutinin inhibition (HAI) assays largely depend upon RBS targeting antibodies to block red blood cell (RBC) agglutination. Influenza vaccine efficacy and regulatory approval depends on the HAI assay although it only captures a limited subset of serum antibodies. To gain a fuller understanding of the immune response elicited by our HA nanoparticle vaccine with Matrix-M adjuvant, we used the RBS A2.91.3 and VE subdomain A2.4.1 antibodies to characterize the antibody response in a pre-clinical ferret study [22] (Figure 7A,B). Immune naïve ferrets (*n* = 10/group) were immunized twice at a three week interval with trivalent nanoparticle influenza vaccine (tNIV, 15 μg/strain + Matrix-M), Fluzone HD (IIV3-HD, 60 μg/strain), Fluzone quadrivalent (IIV, 15 μg/strain), or placebo (PBS) as previously described [22]. Sera from immunized animals from day zero (pre-immunization) and day 42 (post-immunization) were probed for the presence of antibodies that could compete for binding to the homologous H3N2 strain A/Hong Kong/4801/2014 HA nanoparticles using an Octet (bio-layer interferometry) assay. Ferrets immunized with tNIV produced significantly more antibodies that compete with RBS A2.91.3 (Figure 7A) and VE subdomain A2.4.1 (Figure 7B) than either comparator vaccine (*p* < 0.0001). Next the sera from sixteen individuals each chosen randomly from a phase 1 clinical trial (ClinicalTrials.gov number, NCT03293498 [23]) of older adults were analyzed for competing antibodies to head domain antibodies A2.91.3 and A2.4.1 and stem domain antibody CR8020 [15,23]. The fold-rise of competing antibodies from day zero (pre-immunization) were compared to day 21 (post-immunization) for each individual in the vaccination groups. Immunization with tNIV produced a significantly higher fold-rise of antibodies that compete with the head antibodies RBS A2.91.3 (Figure 7C) and VE subdomain A2.4.1 (Figure 7D) than the comparator licensed vaccine (*p* < 0.05). Although there was a two-fold increase in mean fold-rise of competing antibodies to the stem CR8020 site (tNIV = 2.715 versus IIV3-HD = 1.286) the difference was not statistically significant. 

## 4. Discussion

In both pre-clinical and early clinical trial testing of our trivalent HA nanoparticle vaccine, we observed broader neutralization and improved HAI titers compared to a licensed influenza vaccine when testing historical H3N2 strains from the past decade [22,23]. Thus, in this work, two head domain antibodies, A2.91.3 and A2.4.1, covering distinct antigenic sites were characterized and used to evaluate the vaccine-induced serum antibodies. The RBS antibody A2.91.3 binds the 190-helix component of the immunodominant antigenic site B while the VE subdomain antibody A2.4.1 recognizes the highly conserved antigenic site C [44]. Both of these neutralizing head domain antibodies recognized contemporary circulating strains across various subclades that contained mutations in both the dominant antigenic site B and less pressured antigenic sites. In particular, VE subdomain antibody A2.4.1 bound all tested strains and neutralized all recent and contemporary vaccine strains, demonstrating the value of eliciting antibodies towards more conserved, subdominant antigenic sites. Immunization of naïve ferrets with tNIV produced significantly more A2.91.3 and A2.4.1 competing antibodies than comparator vaccines. In older adult subjects, despite previous exposure, there was still a significant fold-rise increase in competing antibodies to both head domains compared to Fluzone HD. These results suggest that the adjuvanted HA nanoparticle vaccine produces a broad and robust antibody-mediated immune response.

In development of a vaccine for a virus with significant seasonal antigenic drift such as influenza A(H3N2) strains, it is critical that vaccine-induced antibody responses cover multiple antigenic sites to improve the capability to neutralize divergent circulating strains [9]. Although regulatory approval depends on serum testing using the hemagglutinin inhibition (HAI) assay, this test captures a limited subset of serum antibodies. Ideally, a full characterization of the antibody-mediated immunity in response to influenza immunization could be used to optimize vaccine design. However, limitations in evaluating the entirety of a subject’s antibody repertoire often reduces comprehensive studies to a single patient or small cohort [5,39,41,44,45]. Also the complexity of prior exposure and most importantly the primary exposure of each individual impacts the ability to generalize such limited studies [9]. Ultimately, while influenza A(H3N2) research has resulted in deeper insight into the structure of HA, the highly conserved sites for targeting bnAbs, and even the evolutionary limitations of viral mutations [46], the most important area of focus for vaccination is to elicit a broad and protective immune response that will dramatically improve on the performance of the current licensed seasonal influenza vaccines.

## 5. Conclusions

Recent experience with the rapid evolution of A(H3N2) influenza strains, and the consequent disappointing vaccine efficacy over multiple seasons, highlights the need for vaccine strategies which can provide protection against strains that have evolved beyond those included in the vaccine. A key aspect of future influenza vaccine antigen development, therefore, will be both the retention of the highly efficacious, but also highly strain-specific, immune responses evoked by current vaccines; complemented by induction of responses to highly conserved neutralizing epitopes which may be found not only in the stem, but also the head, of hemagglutinin [9]. Here we demonstrate that influenza HA nanoparticles, delivered with saponin-based Matrix-M™ adjuvant, are capable of eliciting both classical hemagglutination-inhibiting antibodies but also antibodies reactive with multiple broadly cross-reactive epitopes on hemagglutinin.

## Figures and Tables

**Table 1:** Neutralization of recent wild-type and egg-passaged H3N2 strains.

H3N2 Strain	Subclade	Neutralization IC50 (ng/mL)	
		A2.91.3	A2.4.1
A/Victoria/361/2011	3C.1	2	1437
A/Texas/50/2012	3C.1	14	1010
A/Switzerland/9715293/2013	3a	2	314
A/Hong Kong/4801/2014	2a1	0.6	80
A/Hong Kong/4801/2014—egg-passaged (N96S, T160K, L194P)	2a1	17	652
A/Singapore/0019/2016	2a1a	4	331
A/Singapore/0019/2016—egg-passaged (T160K, L194P)	2a1a	0.4	146
A/Wisconsin/19/2017	2a2	96	805
A/Wisconsin/19/2017—egg-passaged (T160K, L194P, T203I)	2a2	32	178
A/Kansas/14/2017	3a	427	330
A/Kansas/14/2017—egg-passaged (D190N, N246T)	3a	> 10,000	102

## Supplementary Materials

The following are available online at https://www.mdpi.com/2076-393X/8/1/99/s1. Figure S1: FACS analysis of single point mutations in the bottom of the RBS pocket, Figure S2: Models of A2.91.3 and AHK14 HA used for antibody docking, Figure S3: Binding of A2.91.3 mutants to AHK14 HA, Figure S4: Egg-passaged mutations in AHK14 evaluated for A2.91.3 binding, and Table S1: GISAID accession numbers.

## Abbreviations

AHK14	A/Hong Kong/4801/2014
BLI	biolayer interferometry
bnAbs	broadly neutralizing antibodies
CAE	competitive antibody equivalents
CDR	complementary determining region
Fab	antigen binding fragment
FACS	fluorescence-activated cell sorting
HA	hemagglutinin
HAI	hemagglutinin inhibition
HC	heavy chain
HD	high dose
LC	light chain
LOD	limit of detection
mAb	monoclonal antibody
MOI	multiplicity of infection
NIV	nanoparticle influenza vaccine
NP	nanoparticle
PBMC	peripheral blood mononuclear cell
RBS	receptor binding site
Sf9	*Spodoptera frugiperda* cells
TEM	transmission electron microscopy
tNIV	trivalent nanoparticle influenza vaccine
VE	vestigial esterase
VL	variable light
WHO	World Health Organization
WT	wild-type
