# Supplementary material for: Influenza Hemagglutinin Nanoparticle Vaccine Elicits Broadly Neutralizing Antibodies against Structurally Distinct Domains of H3N2 HA
Source: Vaccines (Basel). 2020 Feb 22;8(1):99. doi: 10.3390/vaccines8010099 (PMC7157642; doi:10.3390/vaccines8010099)
Supplement: Supplementary file 1 [file vaccines-08-00099-s001.pdf]

vaccines

Article

Influenza hemagglutinin nanoparticle vaccine elicits broadly neutralizing antibodies against structurally distinct domains of H3N2 HA

Alyse D. Portnoff [1], Nita Patel [1], Michael J. Massare [1], Haixia Zhou [1], Jing-Hui Tian [1], Bin Zhou [1], Vivek Shinde [1], Gregory M. Glenn [1] and Gale Smith [1,]*

[1] Novavax, Inc. Gaithersburg, MD, USA; aportnoff@novavax.com (A.D.P.); npatel@novavax.com (N.P.); mmassare@novavax.com (M.J.M.); hzhou@novavax.com (H.Z.); jhtian@novavax.com (JH.T.); bzhou@novavax.com (B.Z.); vshinde@novavax.com (V.S.); gglenn@novavax.com (G.G.)

* Correspondence: gsmith@novavax.com

Received: date; Accepted: date; Published: date

Supplementary Materials:

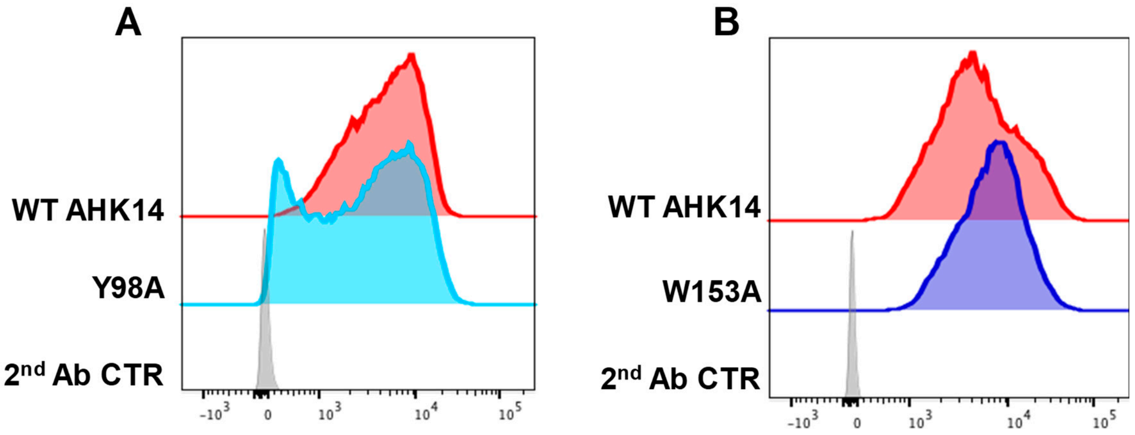

Figure S1. FACS analysis of single point mutations in the bottom of the receptor binding site pocket. Sf9 cells infected with baculovirus carrying recombinant HA mutant genes were co-labeled with stem antibody CR8020 for gating of HA expression and A2.91.3. (**A**) A2.91.3 had reduced binding to AHK14 with the mutation Tyr98Ala in the RBS base. (**B**) A2.91.3 binding was unaffected by the Trp153Ala mutation in the RBS base. Point mutations were tested on separate days with parallel independent baculovirus infections for the wild-type A/Hong Kong/4801/14 control.

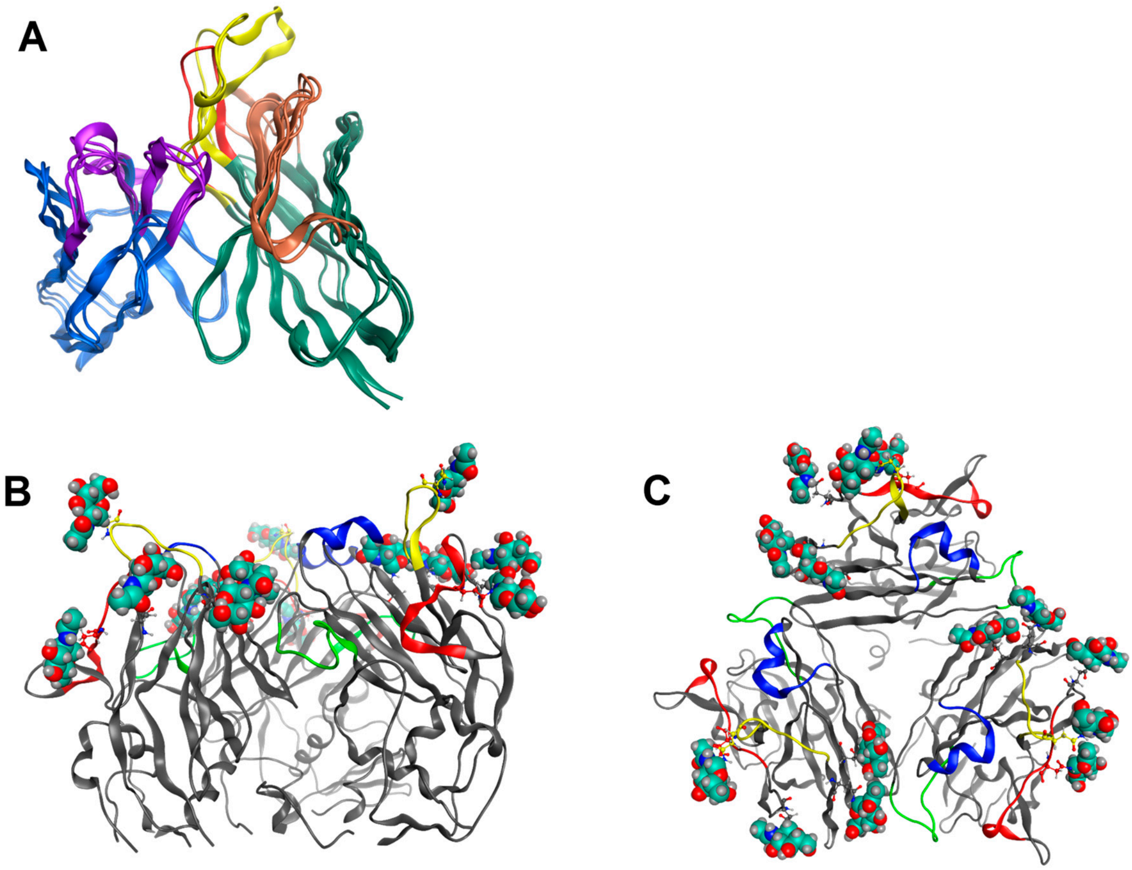

Figure S2. Models of A2.91.3 and AHK14 HA used for antibody docking. (**A**) The variable domain of A2.91.3 compared to crystallized RBS bnAbs F045-092 (PDB 4O5L) and C05 (PDB 4FNL). The Fv model of A2.91.3 was built using Antibody Modeler in MOE. The long CDR H3 domains of C05 and F045-092 are in yellow compared to the 14 amino acid CDR H3 loop of A2.91.3 in red. (**B**) Protein-protein docking was performed with a single GlcNAc at highly conserved glycosylation sites near the RBS on the head domain of HA. (**C**) Top view of glycans modeled on the HA head domain as in (**B**).

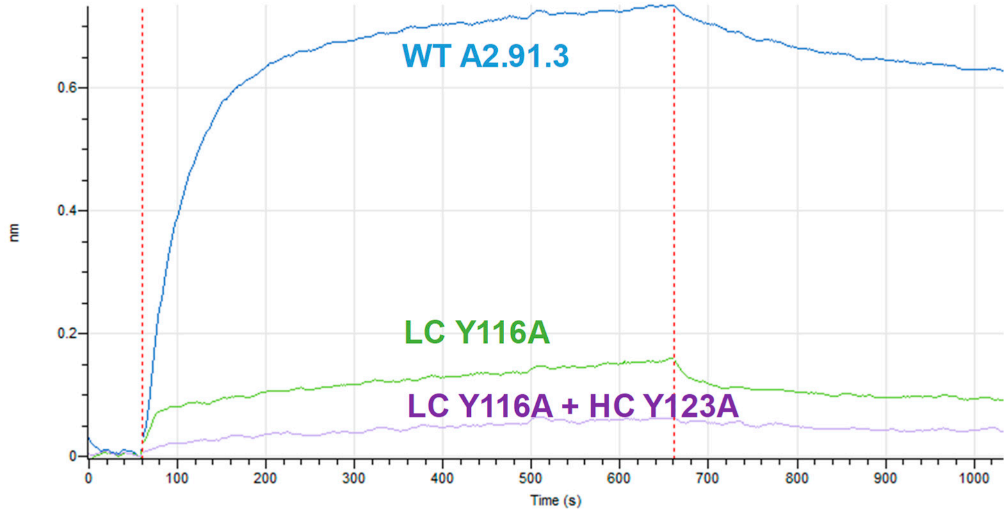

Figure S3. Binding of A2.91.3 mutants to AHK14 HA. AHK14 HA NP was immobilized (0.5 µg/mL) on amine reactive AR2G biosensor tips. The wild-type A2.91.3 RBS antibody (5 µg/mL) bound tightly compared to a single point mutant in the light-chain CDR3 Y116A and a double mutant LC Y116A with heavy chain CDR3 Y123A that had no binding.

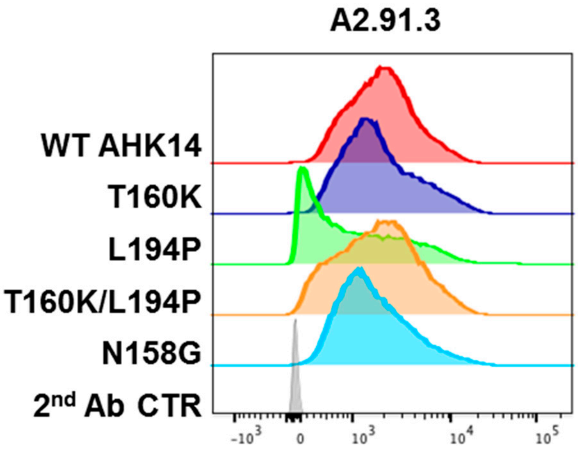

Figure S4. Egg-passaged mutations in AHK14 HA evaluated for A2.91.3 binding. FACS analysis of Sf9 cells infected with baculovirus carrying recombinant HA with egg-derived single and double mutations. Single point mutants T160K and N158G result in a loss of glycosylation at N158 that did not alter A2.91.3 binding. The egg-derived single point mutation L194P reduced binding to A2.91.3 but when combined with the egg-derived T160K mutation (T160K/L194P) recovered binding to A2.91.3.

Table S1: GISAID accession numbers

Strain	Subclade	GISAID accession number
A/Victoria/361/2011	3C.1	EPI349103
A/Texas/50/2012	3C.1	EPI398417
A/Switzerland/9715293/2013	3a	EPI614444
A/Hong Kong/4801/2014	2a	EPI834581
A/Singapore/INFIMH-16-0019/2016	2a1a	EPI780183
A/Iowa/02/2018	2a1b	EPI1174218
A/Pennsylvania/44/2018	2a1b	EPI1205664
A/Switzerland/8060/2017	2a2	EPI1154878
A/Wisconsin/19/2017	2a2	EPI925601
A/Delaware/16/2018	2a2	EPI1206123
A/Iowa/25/2017	2a3	EPI1056984
A/Perth/332/2017	2a4	EPI1153083
A/Kansas/14/2017	3a	EPI1146345
A/Idaho/13/2018	3a	EPI1245228
A/Pennsylvania/78/2018	3a	EPI1245372
A/Hong Kong/4801/2014 egg-passaged	2a	EPI614437
A/Singapore/INFIMH-16-0019/2016 egg-passaged	2a1a	EPI1047604
A/Wisconsin/19/2017 egg-passaged	2a2	EPI925601
A/Kansas/14/2017 egg-passaged	3a	EPI1261067
